# Progress in genome sequencing will accelerate molecular breeding in cotton (*Gossypium* spp.)

**DOI:** 10.1007/s13205-016-0534-3

**Published:** 2016-10-07

**Authors:** Rong Yan, Chengzhen Liang, Zhigang Meng, Waqas Malik, Tao Zhu, Xuefeng Zong, Sandui Guo, Rui Zhang

**Affiliations:** 1 College of Agronomy and Biotechnology, Key Laboratory of Eco-environments in Three Gorges Reservoir Region, Engineering Research Center of South Upland Agriculture, Ministry of Education, Southwest University, Chongqing, 400715 China; 2 Biotechnology Research Institute, National Key Facility for Crop Gene Resources and Genetic Improvement, Chinese Academy of Agricultural Sciences, Beijing, 100081 China; 3 Department of Plant Breeding and Genetics, Bahauddin Zakariya University, Multan, Pakistan

**Keywords:** Genomes, Functional genomics, Agronomic traits, Molecular breeding, Cotton

## Abstract

Cotton (*Gossypium* spp.) is the single most important spinning fiber that has economic significance worldwide. Cotton is one of the most value-added crops and an excellent model system for the analysis of polyploidization and cell development. Thus, the Cotton Genome Consortium has made rapid and significant progress in whole genome sequencing studies in the last decade. Developments in cotton genome sequencing and assembly provide powerful tools for dissecting the genetic and molecular bases of agronomically important traits and establishing regulatory networks on these processes, which leads to molecular breeding. Here, we briefly review these advances, emphasizing their implications in the genetic improvement of cotton with a particular focus on fiber quality and yield. Moreover, major progresses in chloroplast and mitochondrial genomes have also been summarized.

## Introduction

Cotton is a globally important natural fiber and oilseed crop of crucial economic significance (Chen et al. 2007). The unique history of domestication, special fiber structure, and abundant genetic resources make cotton an excellent model system to study polyploidization, cell elongation, and cell wall biosynthesis (Ruan et al. 2003; Qin and Zhu 2011; Wang et al. 2004; Shan et al. 2014). In the last 20 years, biologists and cotton breeders have made rapid and impressive progress in breeding insect-resistant and herbicide-resistant genetically modified (GM) cotton varieties (Guo et al. 2015; Yu et al. 2015; Malik et al. 2015). However, slow progress has been made in genetic improvements of fiber yield and quality, seed modification for food and feed, and cultivation of an ideal cotton architecture for mechanical harvesting. The successful implementation of *Arabidopsis* and rice genome projects has paved the way for consortium-based cotton genome research. The availability of these well-established genome sequences has expedited the progress of cotton functional genomics, improved the understanding of the underlying genetic bases of important agronomic traits, and eventually will apply in cotton molecular breeding. Thus, to sequence cotton genomes, the Cotton Genome Consortium was launched in 2007 (Chen et al. 2007). Recently, many publications have provided draft genome sequences of *Gossypium*, which have implications for genetic improvements and genomics-based breeding (Zhang et al. 2015; Wang et al. 2012a; Liu et al. 2015; Li et al. 2014, 2015).

## Genome sequence of *Gossypium* provides genomic resources for genetic improvement

The *Gossypium* genus constitutes six tetraploid ((A_*t*_D_*t*_)_1_ to (A_*t*_D_*t*_)_5_, where ‘t’ indicates the tetraploid, 2*n* = 4*x* = 52) and at least 46 diploid (2*n* = 2*x* = 26) species, which are believed to have evolved from a common ancestor approximately 5–10 million years ago (MYA) (Grover et al. 2008; Chen et al. 2007). According to their evolutionary relationships and geographic distribution, diploid cotton species have been classified into eight genomic groups (A-G and K) (Cao 2015; Page et al. 2013). They share the same chromosome number (*n* = 13) and have significant genomic synteny with each other (Rong et al. 2004; Desai et al. 2006). Cultivated tetraploid cotton originated approximately 1–2 MYA via hybridization and subsequent allopolyploidization between the native D-genome and after transoceanic dispersion of the A-genome ancestor to the New World (Wendel 1989; Paterson and Wendel 2015). Domestication and artificial selection resulted in two major cultivated tetraploid cotton species, *G. hirsutum* and *G. barbadense*, that have higher fiber yields and quality than improved diploids (Reinisch et al. 1994; Sunilkumar et al. 2006). The recently released genome sequences of two diploid progenitor species, *G. raimondii* and *G. arboretum*, have provided pivotal insight into cotton evolution and the dynamics of genome structures. Additionally, the assembly of two tetraploids, *G. hirsutum* and *G. barbadense*, has opened new avenues for revealing the allotetraploid formation and molecular regulatory mechanisms for some important traits, particularly fiber development.

## *G. raimondii*

For the long-term goal of sequencing cotton genomes, *G. raimondii* (accession *D*_5–3_, DD), a putative contributor of the D-subgenome, was prioritized by the worldwide cotton community. The integrated genetic-physical map of *G. raimondii*, which was reported in 2010, provided important information for future assembly and for validating the reference sequence (Lin et al. 2010). Two synchronous but independent studies that reported the draft genomes of *G. raimondii* were released in 2012 (Wang et al. 2012a; Paterson et al. 2012). Wang and colleagues found that over 73 % of the 78.7-Gb paired-end Illumina reads that covered 103.6-fold of the 775.2-Mb assembled *G. raimondii* genome were anchored to 13 euchromosomes (Table 1). The genome contained 40,976 predicted protein-coding genes, and 92.2 % of them were confirmed by transcriptome sequencing data, which demonstrated the high accuracy of the gene predictions. A total of 13 pseudomolecules that had 737.8 Mb, 37,505 genes, and 77,267 protein-coding transcripts were annotated (Wang et al. 2012a). Paterson and colleagues also performed a genome assembly of *G. raimondii* Ulbr with approximately eight longer N50 scaffolds and that were oriented to 98.3 % of the genome (Paterson et al. 2012). Further, the *Gossypium* genus is the only sequenced plant species that contains an actual *CDN1* gene family for gossypol biosynthesis. Importantly, many gene families, such as Sus (sucrose synthase), KCS (3-ketoacyl-CoA synthase), bHLH, and MYB, are expressed predominantly in *G. hirsutum* ovules, but there are low levels of transcript in the ovules of *G. raimondii* (Wang et al. 2012a). Additionally, Paterson and colleagues found abundant mutation hotspots in the A-subgenome lineage within the fiber-related quantitative trait locus (QTLs) in tetraploid cotton, which may affect multiple fiber traits. Taken together, these studies imply that some of these genes may act as important regulators that are responsible for fiber cell initiation and elongation, and changes in gene-coordinated expression during domestication is an important contribution to cotton fiber development. Dissecting the *G. raimondii* genome is a milestone not only because of its templated significance for analyzing largely parallel re-sequencing data from tetraploids but also because of its importance for tracing the origin of genome segments and homologous genes in tetraploid cotton.

**Table 1:** A summary annotation of *Gossypium* genomes, major genes, and agronomically important traits in different cotton species

	*G. raimondii*	*G. arboreum*	*G. hirsutum*	*G. barbadense*
Variety	*D5*-*3*	*Shixiya1*	*TM*-*1*	*Xinhai2&3*-*79*
Chromosome number	26	26	52	52
Chromosomal pattern	DD	AA	(A_*t*_D_*t*_)_1_	(A_*t*_D_*t*_)_2_
Genomes size (Mb)	775.2/737.8	1694	2173	2470/2570
Genes annotated	40,976/37,505	41,330	76,943	77,526/80,876
LTR size (Mb)	348/391	1145	1471	N/A
Number of LTR-retros	2992/2345	8620	8624	8436
Retrotransposon activity	Stabilization	Very activation	Activation	Activation
Fiber length	No	Short (1.3–1.5 cm)	Long (>3 cm)	Extra-long
*Verticillium* resistance	Resistant	Susceptible	Resistant	Resistant
Reference	Paterson et al. (2012) and Wang et al. (2012a)	Li et al. (2014)	Li et al. (2015) and Zhang et al. (2015)	Liu et al. (2015) and Yuan et al. (2015)

*t* tetraploid

## *G. arboreum*

Both the A- and D-genome diploid species of spinnable fibers are produced by *G. arboreum* (AA), which are planted on a small scale, whereas *G. raimondii* hosts some agronomically important traits (Li et al. 2014; Malik et al. 2014). Two years after the publication of the *G. raimondii* genome, the same researchers sequenced and assembled the genome of the cultivated *G. arboreum*, *Shixiya1* (Li et al. 2014). The quantity of repetitive sequence, including long terminal repeat (LTR)-type retrotransposons, increased from approximately 348 Mb in *G. raimondii* to 1145 Mb in *G. arboreum*, which contributed to the formation of the double-sized *G. arboreum* genome relative to *G. raimondii*. However, the amount of protein-coding genes in these two species was highly conserved. Furthermore, many syntenic blocks were observed between the two diploid species (Table 1). Strikingly, the *ACO* (1-aminoyclo-propane-1-carboxylic acid oxidase) gene family, which is involved in the last rate-limiting step in ethylene biosynthesis, exhibited a greater variation between the promoter regions of *G. arboreum* and *G. raimondii*. This may be pivotally related to the cotton fiber development. In addition, significant qualitative differences in the TN and TNL subfamilies of NBS-encoding type *R* gene transcripts were observed in *G. arboreum* compared with *G. raimondii*. The rapid expansion and contraction of these *R* genes in different cotton species may be responsible for difference resistances to *Verticillium dahlia*. These valuable cotton genes provide a more effective tool for high-yield and disease-resistant genetic engineering.

## *G. hirsutum*

Upland cotton, *G. hirsutum* (A_*t*_D_*t*_)_1_, is widely cultivated in over 80 countries in approximately 33 million ha (5 % of the arable land on earth) (Shan et al. 2014) and it provides more than 90 % of the world’s raw cotton fiber. The annual global economic benefits for the textile industry are approximately 630 billion US$ (Cao 2015; Chen et al. 2007). Compared to diploid cotton species, *G. hirsutum* exhibited significant differences in both plant morphology and economic characteristics, implying that rigorous natural and artificial selection has occurred during evolution. Using the sequence base of two progenitor species, two synchronous but independent cotton genome teams simultaneously completed the genome sequence of the same allotetraploid cultivar, Texas Marker Stock (TM)-1, using second-generation high-throughput sequencing technology that was assisted by traditional paired bacterial artificial chromosome (BAC)-end sequences (Zhang et al. 2015; Li et al. 2015). Despite several differences between the two genomes, such as the number of annotated genes and abundance of retrotransposons and DNA transposons, there are several reasons to convince scientists that the assembly and analyses of the allotetraploid upland cotton genome will begin a new era in functional genomics and marker-assisted breeding (Table 1). First, upland cotton genomes provide not only insights into cotton structural genomics and evolution but also are an important clue for dissecting allopolyploid formation. Second, widespread sharing of *G. hirsutum* genomes will provide new abilities to screen key target genes that are important for fiber development, seed formation, and cotton plant architecture. This will further unravel the molecular mechanisms that underlie these processes. Third, these genomes provide a valuable template for genotyping, phenotyping, and genome-wide association studies that aim to identify important targets of regulators and accelerate cotton crop improvement. Finally, combined with the increased insight, these genomic data and resources will gradually facilitate genomic-assisted cotton breeding from theory to practice. However, understanding whole cotton genomes will initiate a new way of breeding superior cotton cultivars.

## *G. barbadense*

Sea island cotton, *G. barbadense* (A_*t*_D_*t*_)_2_, contributes to approximately 5 % of the annual world cotton production and is famous for its high-quality extra-long fiber for the production of high-grade textiles (Wang et al. 2012b; Reinisch et al. 1994). The quality of fiber is largely dependent on three key factors: fiber length, strength, and fineness. The recently released genome sequences of *G. barbadense*, Xinhai21 (Liu et al. 2015) and 3-79 (Yuan et al. 2015), provided important insight into the molecular mechanisms that are required for superior quality fiber development. Liu and colleagues found that among the 77,526 predicted protein-coding genes of *G. barbadense*, 2483 and 1879 genes are highly and specifically expressed in the fibers and ovules, respectively (Table 1) (Liu et al. 2015). Similarly, Yuan and colleagues identified 708 and 425 homologous gene pairs that are specifically or preferentially expressed in the fiber development stages among the 80,876 predicted protein-coding genes, respectively (Table 1) (Yuan et al. 2015). Interestingly, 58 % of the fiber elongation-related genes and 67 % of the secondary cell wall synthesis-related genes were exclusively either At-biased or Dt-biased (Yuan et al. 2015). These phenomena were also observed in Liu’s studies (Liu et al. 2015). For example, a group of positive cell elongation regulators, Paclobutrazol Resistance (PRE), are significantly A-subgenome tendentious and fiber specific, but the homolog transcripts in the D-subgenome were undetectable, which further supports the origin of the spinnable fiber to be from the A-genome. The expansion, translocation, and subsequent selection of the PRE genes in *G. barbadense* suggested a genetic factor that is responsible for the rate and duration of fiber elongation-determined. Additionally, two independent studies found that compared with *G.hirsutum*, several genes of cellulose synthases (CesAs), such as *CesA4*, *CesA7*, and *CesA8*, underlie secondary cell wall biosynthesis and affect fiber strength and fineness and exhibited a laggard but more significant up-regulation in developing fiber cells of *G. barbadense*. Functional allocation of CesA members conferred extended duration of the elongation stage and highly active secondary wall deposition during the course of extra-long fiber development. Overall, this draft sequence provides valuable information regarding the genes involved in cotton fiber development, and sequencing of the *G. barbadense* genome will facilitate breeding practices aimed at superior fiber quality.

## Ultra-dense genetic linkage maps serve as a valuable tool for cotton genetic research and mark-assisted breeding

Considering the obvious differences in plant characteristics, interspecific crosses between *G. hirsutum* and *G. barbadense* have been widely used for constructing genetic linkage maps, gene cloning, and QTLs that correspond to important agronomic traits. Prior to the release of the four draft sequences of *Gossypium* genome, limited ultra-precision genetic maps were the primary bottleneck that prevented deeper genetic research and breeding of cotton varieties that have a high-yield and extra-long fibers. A comprehensive study was conducted by Wang and colleagues to review the cotton genome structure, and a total of 4,999,048 single nucleotide polymorphisms (SNPs) were detected in 59 interspecific F2 individuals, and two parents, *G. hirsutum* acc. TM-1 *and G. barbadense* cv. Hai7124 (Wang et al. 2015a). Genomic analysis revealed that these SNPs covered a total of 4042 cM but were distributed disproportionally in 26 allotetraploid cotton linkage groups. Meanwhile, another high-density genetic map was developed using simple sequence repeat (SSR) and SNP markers that were derived from an interspecific cross between the sequenced cotton varieties, TM-1 and 3-79. Using the genotype 186 recombinant inbred lines (RILs), 2,027 loci were mapped to 26 chromosomes with an average marker interval of 1.63 cM (Yu et al. 2012). In addition, a high-resolution intraspecific linkage genetic map between the two upland cotton cultivars, Acala Prema and 86-1, was constructed using restriction site-associated DNA (RAD) sequencing technology (Wang et al. 2015b). Combined with the two interspecific maps, these studies indicated a high degree of collinearity in the marker order between these two maps. These high-precision genetic and physical maps provide a useful tool to reveal genome rearrangements, to rectify the anomalies in the cotton reference genome assemblies, to merge scaffolds into pseudomolecules that correspond to the chromosomes, and to detect centromeric regions in allotetraploid cottons. These high-precision genetic maps will not only provide a valuable genomic resource to enhance our understanding of polypoid genome structure, evolution, and tagging of the genome-wide linkage disequilibrium but will also open new avenues for marker-assisted selection in cotton and cloning of QTLs for valued traits.

## The complete chloroplast and mitochondrial genomes of *Gossypium* provide insight into the cytoplasmic–nuclear interaction

Chloroplast (cp) and mitochondria (mt) DNA are maternally inherited. A low rate of nucleotide variation in the cp and mt genomes is a compelling reason for their use in plant evolutionary analysis. Currently available evidence indicates that cytoplasmic-nuclear incompatibility causes cytoplasmic male sterility (CMS) in plants (Wu et al. 2015). Chloroplast genetic engineering provides a new strategy to confer plant herbicide and insect resistance and abiotic tolerance (Jin and Daniell 2015).

The cp genome sequences of *G. hirsutum* and *G. barbadense* were published in 2006 (Lee et al. 2006; Ibrahim et al. 2006). Soon afterwards, large-scale cp genome sequence variations among the different cotton species were investigated by re-sequencing two A-genome lineages, two D-genome lineages, two *G. hirsutum*, three *G. barbadense*, and three wild allotetraploid cottons accessions (Xu et al. 2012). Combined with the aforementioned two cp genomes, the complete length of 14 cp genomes varied from 159,959 to 160,433 with the same set of 112 unique genes and 19 duplicated genes. A general comparison indicated that there was a high similarity among the different cotton cp genomes regarding genome size, genome structure, gene number, and gene order. However, the boundary junctions between the inverted repeat (IR) and small single copy (SSC) regions were different. The lower proportion of the divergence in the protein-coding regions suggested that natural variations in the major part of the cp genome that are responsible for cotton co-evolution and domestication should be attributed to the intergenic regions. The completion of the *Gossypium* cp genome sequencing provided vital information for both fundamental theoretical research and practical application. In particular, the available cotton cp sequences provided useful information for chloroplast genetic engineering to confer beneficial agronomic traits and serve as bioreactors for valuable bio-production.

The mitochondrion is the core manufacturer of cellular ATP in eukaryotes. In the practical breeding programs, the detrimental mt-nuclear interaction induces plant CMS, enabling ‘three-line’ hybrid development to increase plant yield. To gain insights into the *Gossypium* mt genomes and structure, Hua’s group first completed the sequencing of the upland cotton mt genome, and they identified a total of 35 coding-protein genes, 4rRNA genes, and 29 tRNA genes (Liu et al. 2013). Like cp, the cotton mt genome possesses the majority of the same characteristics as other higher plants. The existence and conservation of syntenic gene clusters, intergenic sequences, and genetic contents in the cotton mt genome indicated that the evolution of mt genomes is largely congruent with plant taxonomy. Importantly, the research on the cotton mt genome will not only help us to identify the effect of male-sterile genes on the stamen in the male-sterile line but also provide insights into the mechanism of the nucleo-cytoplasmic interaction in plants.

## What is the future for breeding superior cotton varieties based on these draft genome sequences?

**Fig. 1:**
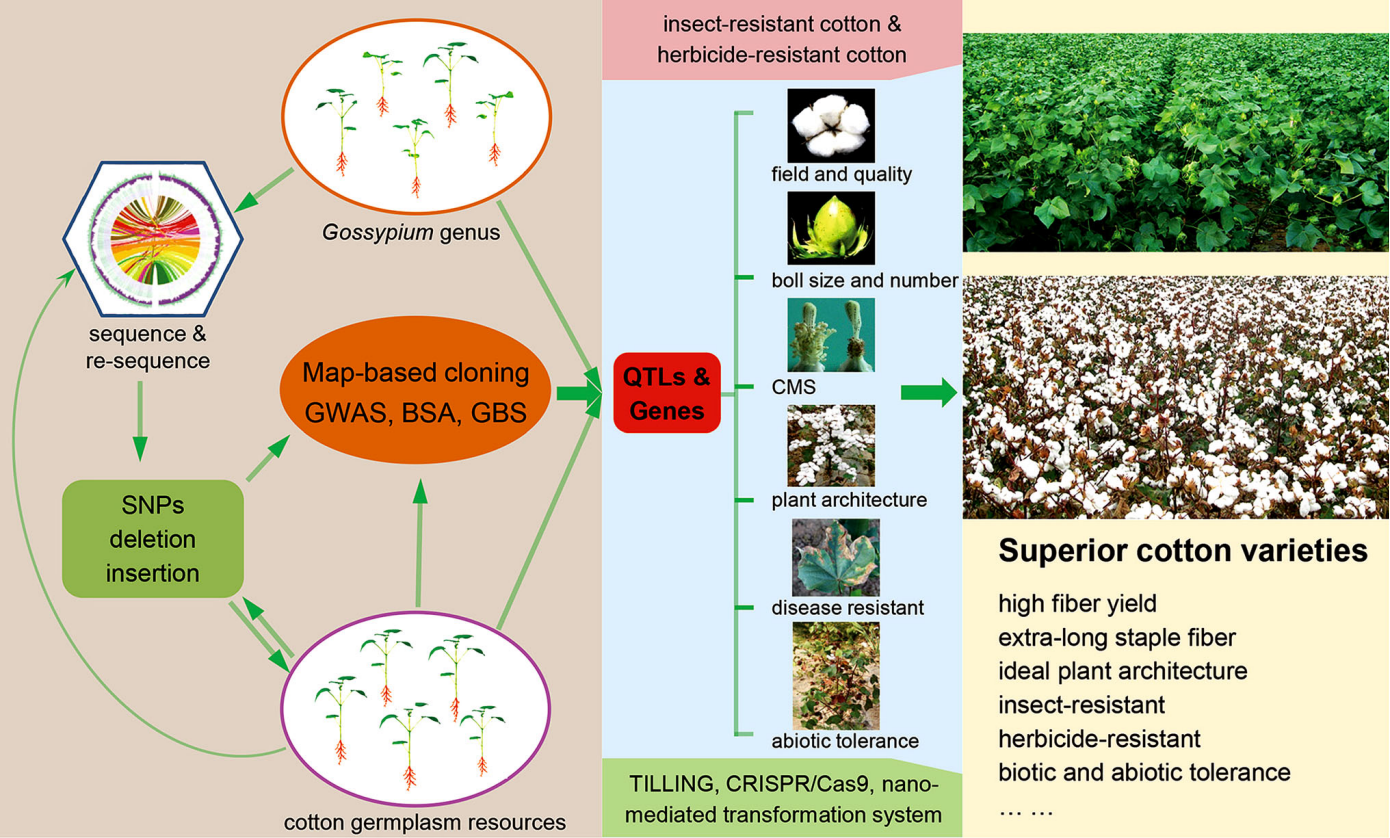
A scheme of the sequential research processes of genome-based breeding from whole genome sequencing to practical engineering in cotton. *SNPs* single nucleotide polymorphisms, *GWAS* genome-wide association study, *BSA* bulked segregant analysis, *GBS* genotyping-by-sequencing, *CMS* cytoplasmic male sterility, *TILLING* targeting induced local lesions in genomes. The diagram of the genomes is reproduced from Li et al. (2014)

Whole genome sequencing has paved the way for cotton improvement from the bench to molecular breeding. The implementation of genome-assisted molecular breeding must include three coordinated steps (Fig. 1). First, the development of an efficient system to mine major genes and QTLs for agronomical important traits is required. As demonstrated by recent studies in *Arabidopsis* (Koornneef et al. 2004), rice (Zuo and Li 2014; Huang and Han 2014), and maize (Yang et al. 2013; Jiao et al. 2012), because of the rapid advances in whole genome sequencing, genetic mapping of mutations has been revolutionized by map-based cloning, bulk segregant analysis (BSA), genotyping-by-sequencing (GBS), and genome-wide association studies (GWAS). There is no doubt that sequenced-based high-precision molecular markers of intraspecific and interspecific populations of cotton will play a crucial role in map-based cloning QTLs and major genes. Unlike Arabidopsis and rice, the progress of mutant population generation has not occurred at the same pace because of several factors, including complex and huge genome and the low efficiency of genetic transformation. Therefore, in the coming years, the dissection and cloning of complex QTLs control phenotypic variations, especially agronomically important traits within the germplasm, will be a major focus and great challenge for cotton genetic studies and molecular breeding. The completion of cotton whole genome sequencing will enable rapid identification of SNPs or small indels on a genome-wide and population-wide scale (Hulse-Kemp et al. 2015). Consequently, in light of model plant results, whole genome genotyping of hundreds of cotton varieties with well-characterized phenotypes will be feasible for mapping important agronomic traits. Second, the development of an efficient system to identify genes and QTLs that can be manipulated is required. The forward genetic approach and reverse genetic approach, such as mutants, near-isogenic lines (NILs), and transgenic approach, are essential methods for gene functional analysis. However, in many cases, a complex trait is usually regulated by multiple loci, whereas a single gene is also directly or indirectly involved in controlling different phenotypes. Therefore, experimental studies on the biological functions of genes are facing enormous challenges. Recently, several scientific research groups have been in the process of developing a multi-scale crop system (Long et al. 2015; Chu 2015; Chew et al. 2014), such as the ePlant model (Zhu et al. 2013), which can be used for high-throughput and systematic association studies of multiple loci and complex traits. Finally, the development of an efficient system for molecular breeding by multiple function modules is necessary. The widespread use of recently developed genome editing technologies, such as the clustered regularly interspaced short palindromic repeats associated system (CRISPR-Cas9) (Shan et al. 2013), transcription activator-like effector nucleases (TALENs) (Miller et al. 2011), and target-induced local lesions in genomes (TILLING) (McCallum et al. 2000), as well as the nanocarrier-mediated genetic transformation system, provide excellent alternatives for cotton improvement. The studies of genome-based molecular breeding in rice and maize should be effectively utilized to accelerate cotton breeding. However, unlike rice and maize, transgenic cotton, including Bt-toxin-containing cotton, herbicide tolerance, and the stacked traits of insect-resistant and herbicide-tolerant cotton have been successfully commercialized in the past 20 years (Guo et al. 2015; Meng et al. 2015). Thus, scientifically, fast and fluent communication and feedback channels between molecular biologists and cotton breeders need to be established to better guide and facilitate breeding and engineering to produce ideal agronomic cotton traits in the future.

## Challenges and future perspectives

During the past decade, we have witnessed tremendous progress in sequencing and assembly of whole cotton genomes, including two diploid cotton species, two tetraploid cotton species, and several *Gossypium* cp and mt genomes. These achievements have made breakthroughs in several important fields, including the identification and characterization of several important genes or gene families. These achievements have included the regulation of fiber development and gossypol biosynthesis, dissection of signal pathway responses to *Verticillium* wilt resistance and abiotic stresses, and the elucidation of regulatory mechanisms of genome evolution, domestication, and polyploidization. These achievements will serve as a bridge between comparative genomics, functional genomics, and modern cotton breeding. However, compared to *Arabidopsis* and rice, there are still large knowledge gaps regarding the molecular regulation of the basic biological processes that are related to important agronomic traits. These gaps exist because of the difficulty to identify the targets of key regulators underlying these processes and to establish a quick and simple genetic transformation system. Obviously, cloning and functional characterization of more pivotal genes and QTLs that control complex physiological and agronomy traits will be a major challenge for future studies.

The ample quantitative differences in agronomic traits, especially those involved in fiber quality and yield between the intra- and inter-species of *Gossypium*, represent variables that can be improved upon in breeding programs. The accumulation of genomic and functional genomic studies will be multiplied upon in the next generation of cotton cultivars. These data will ideally result in extra-long fibers, high fiber yields, and improved plant architectures that are suitable for mechanical harvesting. In addition, knowledge from systems biology approaches, especially from different omics studies, such as transcriptomics, proteomics, and metabolomics, will allow us to dissect the related traits in more detail and explore the regulation pathways and networks governing these traits. Therefore, there is no doubt that with the continuous development of cotton genome sequencing and genome editing technologies, as well as improved transgenic technology and use of marker-assisted selection, we can innovate germplasms and cotton breeding in effective ways.
